# Enchondroma of Anterior Maxilla: A Rare Entity

**DOI:** 10.7759/cureus.32834

**Published:** 2022-12-22

**Authors:** David Tyro, G Madhusudhana Rao, Navaljeet Kaur, Aswani Sreenivasan, Subramanya S Sharma

**Affiliations:** 1 Oral Surgery, Army College of Dental Sciences, Secunderabad, IND; 2 Oral and Maxillofacial Surgery, Army College of Dental Sciences, Secunderabad, IND

**Keywords:** head and neck pathologies, hyaline cartilage, chondroma, cartilaginous tumour, case report, enchondroma

## Abstract

Enchondroma is a benign cartilaginous tumor composed of mature hyaline cartilage. Cartilaginous tumors are detected in a small percentage of cases in the craniofacial region. These tumors may have diverse presentations ranging from a simple enchondroma to a high-grade osteo or chondrosarcoma. In the maxilla, only 1 case of enchondroma has been reported in the literature to date, to the best of the authors' knowledge. Tumor's membranous development attributes to its occurrence usually in the cartilage-bearing areas of the jaws, like the condylar process of the mandible (Meckel’s cartilage). This case report intends to present one case of enchondroma involving the left maxilla, which has no primary cartilage of its own.

## Introduction

Enchondroma is a benign cartilaginous tumor composed of mature hyaline cartilage [1]. Cartilaginous tumors are detected in a small percentage of cases in the craniofacial region. Tumor's membranous development attributes to its occurrence in the cartilage-bearing areas of the jaws, like the condylar process of the mandible (Meckel’s cartilage). This article intends to present the enchondroma of the maxilla that has no primary cartilage of its own. The authors' proposed hypothesis is that nasal septal cartilage, which is closely associated with the maxilla, could be the probable origin of this case.

A benign growth of mature hyaline cartilage known as a chondroma is mainly seen in the extremities (96%), with 72% in the upper limb, 24% in the lower limb, 2% in the head and neck and 2% in the trunk [1].

Depending on their locations, chondromas can be classified as enchondromas which originate from the marrow in the medullary cavity of the bony skeleton, juxtacortical or periosteal chondromas, which originate adjacent to the periosteum below the cortical surface and extra-skeletal or soft tissue chondromas [2].

Enchondromas or central chondromas account for 0.32% of all entities in the craniofacial region, making it quite uncommon [3]. We hereby present an instance of an enchondroma in the anterior maxilla, an occult entity; according to the available literature, only two cases have been reported to date in the same location. This article can serve as a pioneer case for future reference. 

## Case presentation

A 52-year-old female patient reported to our institute with a complaint of swelling in the upper front tooth region for five months and also complains of forwardly displaced upper front teeth. The patient noticed a mild gap in the upper front teeth (canine-premolar) region three years ago, which gradually increased in the following years to the present size. The patient started developing swelling in the upper front tooth region in November 2021, which gradually increased to 3x2cm in size. She also gives a history of displacement of upper front teeth due to the swelling. The swelling was not associated with any local discharge or paresthesia. The medical history was non-contributory.

**Figure 1:**
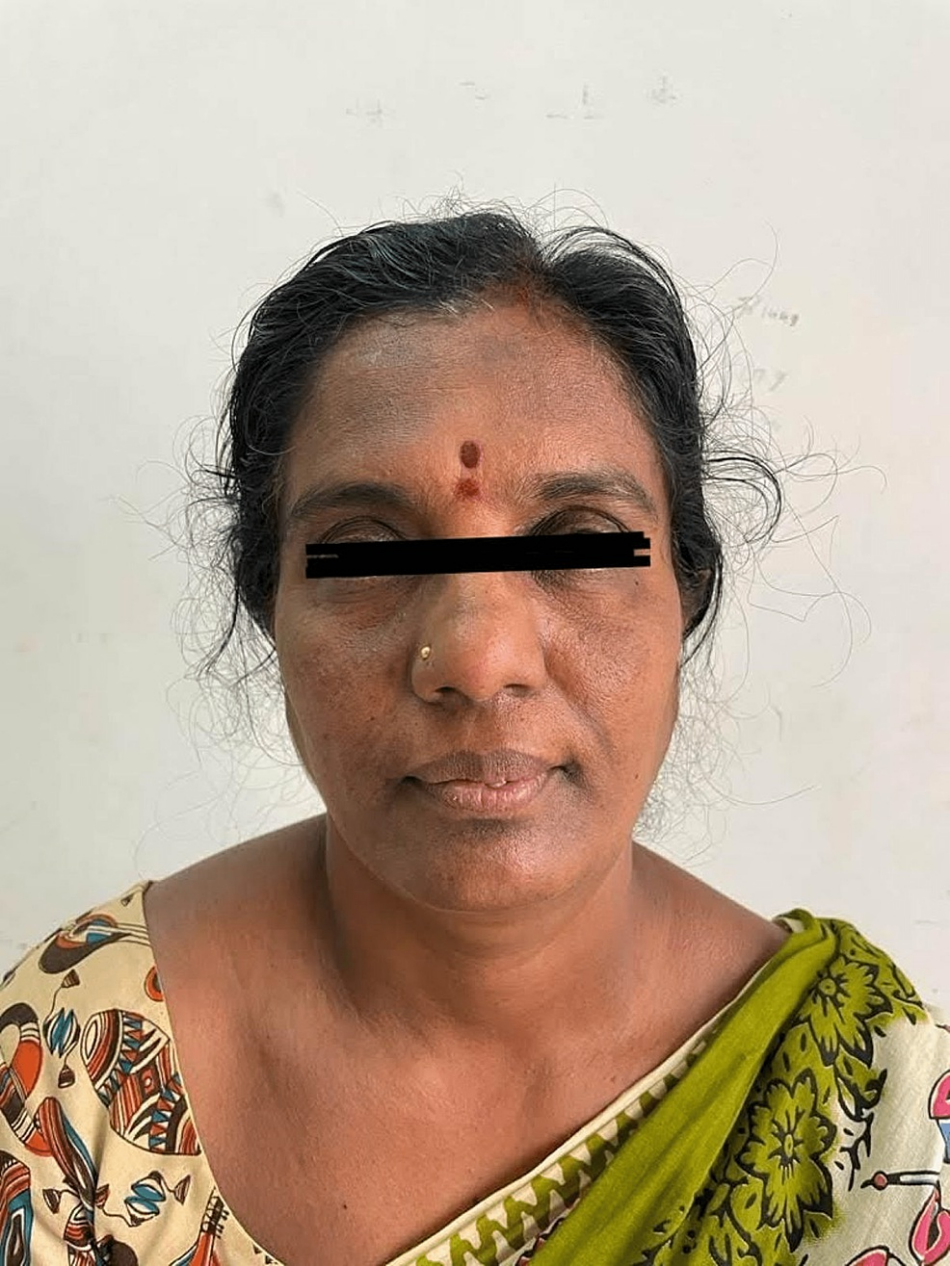
Preoperative frontal profile view depicting the swelling in the left upper front tooth region

**Figure 2:**
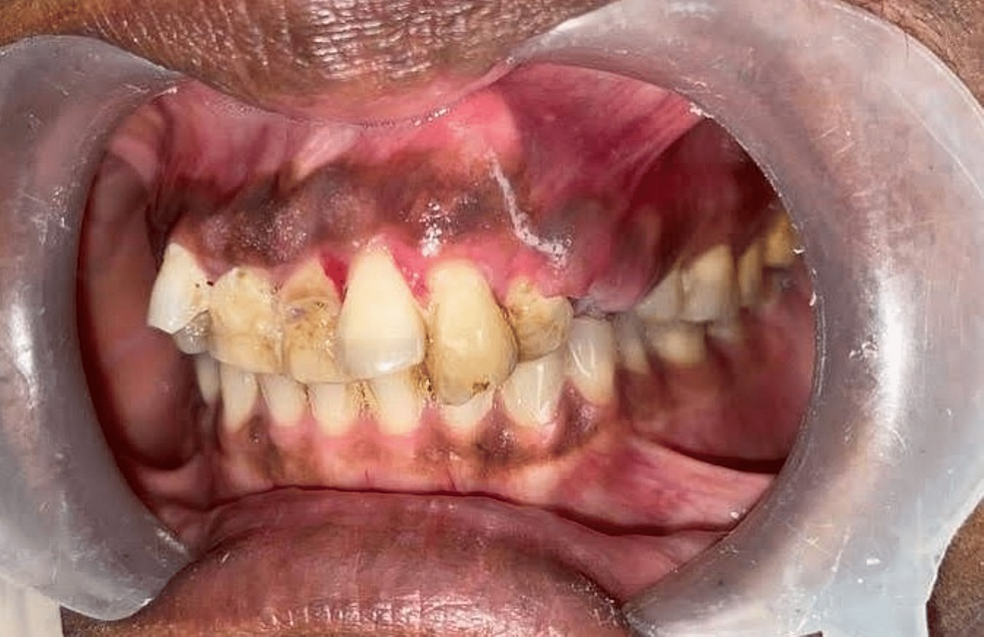
Intraoral picture showing the oval-shaped swelling in the periapical region of 22, 23, and 24, 25.

**Figure 3:**
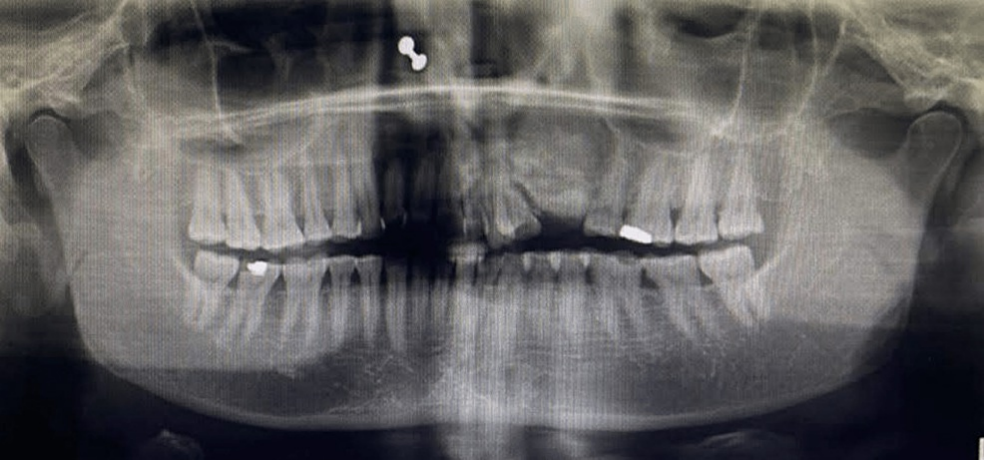
OPG reveals the mixed radiopaque radiolucent lesion with ill-defined borders involving maxillary alveolus extending from 21 to 25 region with a displacement of 23, 24.

**Figure 4:**
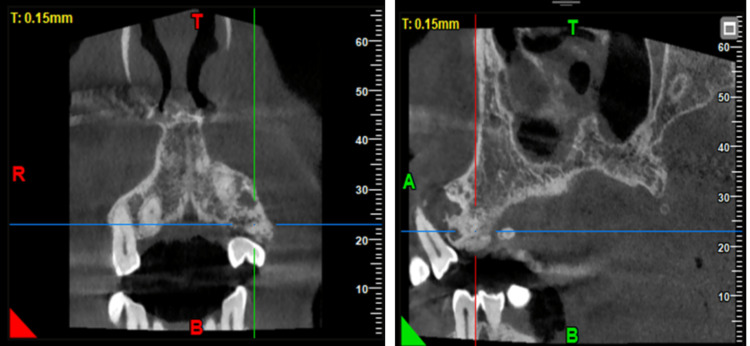
CBCT depicting the mixed radioopaque-radiolucent lesion involving the maxillary alveolus with a displacement of 24.

On clinical examination, there was a mild diffuse swelling present on the left side of the face below the ala of the nose. (Figure 1) Overlying skin appeared normal, and the left ala of the nose was slightly raised. A firm, oval-shaped swelling was found intraorally in the periapical region of teeth 22 and 23, and another oval-shaped swelling was present in the 24 and 25 regions. Labially displaced 21, 22, and 23 with midline shift and spacing existed between 24 and 25, hinting at pathological tooth movement (Figure 2). On radiographical investigation, orthopantomogram (OPG) reveals mixed radio-opaque radiolucent lesions with ill-defined borders involving maxillary alveolus extending from 21 to 25 region with a displacement of 23, 24 (Figures 3, 4).

Since the lesion was a firm, painless, well-circumscribed swelling fibrous dysplasia, ameloblastic fibro-odontoma, complex odontoma, and chondroma were considered differential diagnoses.

Incisional biopsy was performed from the periapical region of 24 and the labial aspect of 22 and 23 under local anesthesia. Hard tissue specimens measuring 0.2 cm and 0.4 cm were obtained, respectively. Histopathological sections showed small cartilaginous fragments displaying a cellular lesion in small lobules comprising of chondrocytes with small bland chondroid cells in lacunar spaces, uniform staining nuclei, no pleomorphism, anaplasia, mitosis, hyperchromasia and multinucleated giant cells. Hence the lesion was diagnosed as enchondroma of the left anterior maxilla.

**Figure 5:**
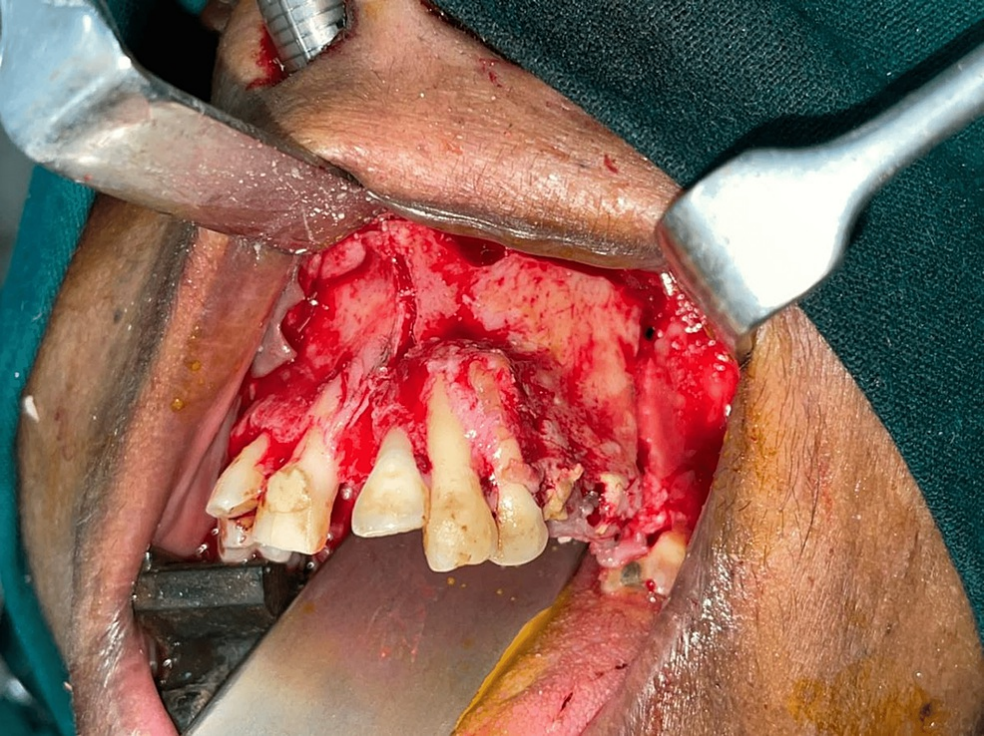
The intraoperative picture with osteotomy cuts

**Figure 6:**
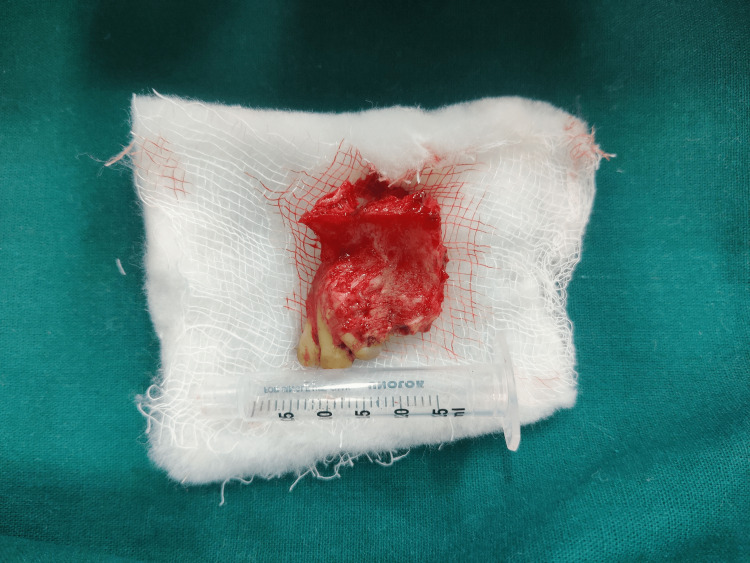
Resected specimen

**Figure 7:**
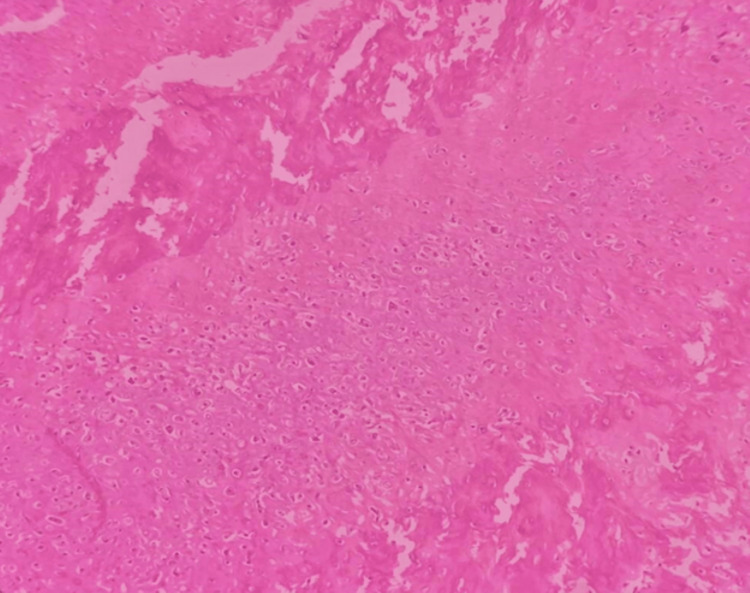
Histopathological sections reveal mature hyalinized stroma with lobules of chondrocytes and band chondroid cells enclosed in lacunar spaces with few tiny spicules of normal bone suggestive of Enchondroma.

The lesion was planned for an en-bloc resection with respect to left maxilla extending from 21 to 25 region with 1 cm peripheral margins under general anesthesia using intra- oral crevicular incision extending from 13 to 27 followed by primary closure using the buccal pad of fat and mucosa (Figures 5, 6). Histopathological sections reveal mature hyalinized stroma (Figure 7).

**Figure 8:**
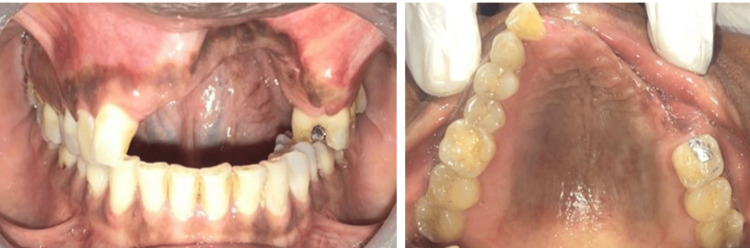
Post-operative intraoral picture at 8 months follow-up

The case is on continuous follow-up for eight months to check for any reoccurrence and is currently undergoing prosthetic rehabilitation (Figure 8).

## Discussion

The benign, cartilaginous enchondromas, first coined by Muller in 1838, are slow-growing and asymptomatic tumors found in the medullary cavity of the bone. Having equal sex predilection most frequently affects long bone and short tubular bones of the hand, and it most frequently occurs in the third or fourth decades of life [4]. Maxillary enchondroma is found in the anterior area, close to the nasal septum and nasal spine. The majority of chondromas in the craniofacial complex emerge from the nasal septum, and ethmoidal complex developed from vestigial cartilaginous remnants [5]. Mandibular chondromas have been observed in the symphysis, body, coronoid process, and mandibular condyle [6].

In a review of 8542 bone tumors conducted by Dahlin and Unni, chondromas account for 2.8% of all tumors and 12% of benign tumors. These tumors' occurrence in non-cartilaginous jaw regions has led some authors to refer to them as hamartomatous growth [7]. However, some researchers have demonstrated that in these situations, abnormal embryonic cell rests and multidirectional differentiation of mesenchymal cells are related to the development of tumors [8].

Enchondromas usually present as painless, slow-growing, and well-confined tumors with an ovoid or a variable convexity. The lesion rarely involves overlying mucosa, and tooth mobility and root resorption are possible [9]. It exhibits two special clinical forms: Olliers disease and Maffucci syndrome.

The radiographic findings of chondromas are not typical. It is possible to see a mottled, amorphous mass that is radiolucent and radiopaque. The lack of cortical damage and soft tissue extension favors a benign diagnosis [10]. A lobular arrangement of hyaline cartilage with well-formed lacunae containing lobules of chondrocytes with small band chondroid cells, regular chondrocytes are characteristic histological features [11]. Typically, mononuclear chondrocytes are seen with hyperchromatic nuclei. Calcifications are occasionally observed in the vacuolar chondroid substance [1].

The distinction between chondroma and chondrosarcoma may be difficult because of overlapping histologic features [9]. Distinguishing chondroma from high-grade chondrosarcoma presents no difficulty. A useful characteristic is the size of the lesion. Most chondromas have been observed to be between 1 and 3 cm, but chondrosarcomas are larger than 5.5 cm. The presence of one or more mitotic figures indicates a high probability of malignancy. Additional manifestations of ongoing, unremitting pain, together with radiological evidence of cortical erosion and soft tissue extension, are ominous indicators of malignancy [9].

Therefore, it has been argued that all symptomatic cartilaginous lesions should be classified as chondrosarcomas and treated accordingly. Many pathologists believe that the presence of a chondroma indicates a potential chondrosarcoma. Due to the possible clinical and histological overlap and consideration that twenty percent (20%) of head and neck chondrosarcomas may be initially diagnosed as benign, the treatment of the enchondroma is a wide, although not radical, excision. The resection with a margin of normal soft tissue and bone is preferred. In a considerable proportion of cases, curettage has led to local recurrence [7]. As the tumour is not radiosensitive, radiotherapy is not recommended. The lesions should be excised with 1-cm peripheral margins, considering the recurrence potential of chondrosarcoma (20%-33%) [7].

Long-term follow-up is crucial. The initial diagnosis should be re-evaluated if recurrence occurs to rule out the possibility of low-grade malignancy [7].

## Conclusions

Due to the dearth of available literature, it is imperative to report such cases. Extreme vigilance should be used from the time of diagnosis to rule out enchondroma due to the potential clinical overlap with fibro-osseous lesions and other benign lesions of the jaws. Enchondroma leads to disruption of structure, compromising the aesthetics and function. Long-term clinical and radiological follow-up is required because of its high propensity for recurrence, as well as the likelihood of malignant change.
